# Alanyl-Glutamine Protects against Lipopolysaccharide-Induced Liver Injury in Mice via Alleviating Oxidative Stress, Inhibiting Inflammation, and Regulating Autophagy

**DOI:** 10.3390/antiox11061070

**Published:** 2022-05-27

**Authors:** Jiaji Hu, Hanglu Ying, Yigang Zheng, Huabin Ma, Long Li, Yufen Zhao

**Affiliations:** 1 Institute of Drug Discovery Technology, Ningbo University, Ningbo 315211, China; hjj0607@126.com (J.H.); 1911074039@nbu.edu.cn (H.Y.); 2111074064@nbu.edu.cn (Y.Z.); mahuabin@nbu.edu.cn (H.M.); zhaoyufen@nbu.edu.cn (Y.Z.); 2 Qian Xuesen Collaborative Research Center of Astrochemistry and Space Life Sciences, Ningbo University, Ningbo 315211, China; 3 State Key Laboratory of Chemical Oncogenomics, Tsinghua Shenzhen International Graduate School, Shenzhen 518055, China

**Keywords:** acute liver injury, alanyl-glutamine, apoptosis, oxidative stress, inflammation

## Abstract

Acute liver injury is a worldwide problem with a high rate of morbidity and mortality, and effective pharmacological therapies are still urgently needed. Alanyl-glutamine (Ala-Gln), a dipeptide formed from L-alanine and L-glutamine, is known as a protective compound that is involved in various tissue injuries, but there are limited reports regarding the effects of Ala-Gln in acute liver injury. This present study aimed to investigate the protective effects of Ala-Gln in lipopolysaccharide (LPS)-induced acute liver injury in mice, with a focus on inflammatory responses and oxidative stress. The acute liver injury induced using LPS (50 μg/kg) and D-galactosamine (D-Gal) (400 mg/kg) stimulation in mice was significantly attenuated after Ala-Gln treatment (500 and 1500 mg/kg), as evidenced by reduced plasma alanine transaminase (ALT) (*p* < 0.01, *p* < 0.001), aspartate transaminase (AST) (*p* < 0.05, *p* < 0.001), and lactate dehydrogenase (LDH) (*p* < 0.01, *p* < 0.001) levels, and accompanied by improved histopathological changes. In addition, LPS/D-Gal-induced hepatic apoptosis was also alleviated by Ala-Gln administration, as shown by a greatly decreased ratio of TUNEL-positive hepatocytes, from approximately 10% to 2%, and markedly reduced protein levels of cleaved caspase-3 (*p* < 0.05, *p* < 0.001) in liver. Moreover, we found that LPS/D-Gal-triggered oxidative stress was suppressed after Ala-Gln treatment, the effect of which might be dependent on the elevation of SOD and GPX activities, and on GSH levels in liver. Interestingly, we observed that Ala-Gln clearly inhibited LPS/D-Gal exposure-induced macrophage accumulation and the production of proinflammatory factors in the liver. Furthermore, Ala-Gln greatly regulated autophagy in the liver in LPS/D-Gal-treated mice. Using RAW264.7 cells, we confirmed the anti-inflammatory role of Ala-Gln-targeting macrophages.

## 1. Introduction

Acute liver injury is a serious clinical syndrome affecting public health worldwide, and is mainly characterized by the destruction of hepatic microarchitecture, apoptotic hepatocytes, a massive inflammatory response, and impaired liver function [1]. Numerous factors could be the stimulators of acute liver injury, such as the hepatitis virus, drugs, and ethanol [2]. At present, there are still few effective therapeutic strategies for the treatment of acute liver injury [1]. Thus, novel therapies and drugs are urgently needed. It has been confirmed that hepatic oxidative stress is a main characteristic of acute liver injury [3]. Several studies have demonstrated that inhibiting oxidative stress could alleviate the development of acute liver injury, indicating that antioxidant agents might be effective strategies for the treatment of acute liver injury [4,5]. Autophagy is a homeostatic degradative process that could clear the misfolded proteins and damaged organelles, and is involved in the progression of various liver diseases [6,7]. Recent findings have suggested that defective autophagy in liver is often be detected during the pathogenesis of acute liver injury, and that the reactivation of autophagy could be an effective therapy for the prevention of acute liver failure [8,9]. Lipopolysaccharide (LPS) is an endotoxin from Gram-negative bacteria that can induce the expression of various inflammatory cytokines and chemokines, including tumor necrosis factor-α (TNF-α), interleukin-1β (IL-1β), and monocyte chemoattractant protein-1 (MCP-1), which subsequently leads to hepatocyte injury [10]. D-galactosamine (D-Gal) is a cytotoxic compound that could enhance LPS-induced hepatotoxicity [11,12]. Therefore, the LPS/D-Gal-induced liver damage model has become a widely used animal model that can closely mimic the clinical symptoms of acute liver injury, for the exploration of the mechanisms and potential hepatoprotective strategies of acute liver failure [11].

Glutamine is the most abundant free amino acid in the human body, and it comprises around 20% of the free amino acids in the blood circulation system. It was proven to play a key role in multiple physical processes, including interorgan nitrogen transport, cellular redox pathways, and the synthesis of glutathione, nucleotide bases and peptides [13,14]. It is noted that glutamine is an insoluble amino acid in aqueous solution and it is unstable after high-pressure disinfection, which largely limits its use in clinical medicine. Recently, it has been reported that dipeptides have a good degree of solubility and stability in water, and they can quickly release free amino acids in the body [15]. The dipeptide L-alanyl-L-glutamine (Ala-Gln), which is a compound of alanine and glutamine via an amide bond, was clinically used as a parenteral nutrition medicine for L-glutamine supplementation in critically ill patients who had suffered from trauma associated with burns, surgery, or sepsis [16]. Many studies have revealed the protective effects of Ala-Gln in the inhibition of inflammatory responses, and in the prevention of various tissue injuries, including ulcerative colitis and ischemia–reperfusion injuries appearing in the heart, liver, or brain [17,18,19,20]. Importantly, several reports have demonstrated that Ala-Gln has protective effects against ischemia–reperfusion injury via the improvement of microcirculation, alleviating inflammation, and increasing GSH levels in liver [21,22,23]. It has been reported that Ala-Gln administration could attenuate tissue damage in LPS-induced lung injury in rats [24]. However, the potential effects of Ala-Gln on endotoxin-induced acute liver injury have not been clearly investigated.

In the present study, we aimed to investigate the effect of Ala-Gln on LPS/D-Gal-induced acute liver injury in C57BL/6 mice. The data indicated that Ala-Gln prevented liver dysfunction induced using LPS/D-Gal via the attenuation of oxidative stress and the suppression of inflammation.

## 2. Materials and Methods

### 2.1. Reagents

The Ala-Gln was provided by our collaborators in Xiamen University, and was synthesized according to a previous method [25]. LPS (L4130), D-Gal (G0500), and other chemicals were obtained from Sigma-Aldrich (Shanghai, China).

### 2.2. Animal Experiments

Male C57BL/6 mice, aged 6–8 weeks old, weighing 21–24 g, were provided by Vital River Laboratory Animal Technology Co., Ltd. (Beijing, China). The mice were maintained in an environment with a controlled temperature (21–23 °C), constant humidity (55–60%), and 12 h light/dark cycles. All animal experiments were approved by the Committee for Animal Research at Ningbo University, and were performed according to the guidelines for the care and use of laboratory animals. All mice were randomly divided into five groups (*n* = 6–8/group) as follows: (i) Control group: mice received an intraperitoneal administration of saline; (ii) AG-control group: mice received an intraperitoneal administration of Ala-Gln (1500 mg/kg); (iii) Model group: mice received an intraperitoneal injection of LPS (50 μg/kg)/D-Gal (400 mg/kg) and saline; (iv) Low-dose AG treatment group: mice received an intraperitoneal administration of Ala-Gln (500 mg/kg) and LPS/D-Gal; and (v) High-dose AG treatment group: mice received an intraperitoneal administration of Ala-Gln (1500 mg/kg) and LPS/D-Gal. Ala-Gln was injected 2 h before the LPS/D-Gal treatment, and the liver and plasma samples were collected 5 h after the LPS/D-Gal injection. The mice were sacrificed under anesthesia using an overdose of isoflurane inhalation, as described previously [26,27].

### 2.3. Histological Studies and IHC Analysis

Liver samples were collected and fixed in 4% paraformaldehyde overnight, then embedded in paraffin and cut into 5μm sections for hematoxylin and eosin (HE) staining. The histological changes were observed under an optical microscope (DM750, Leica, Shanghai, China), and scored according to Suzuki’s criteria, which are detailed in a previous report [11]. For immunohistochemistry staining, liver sections were deparaffinized in xylene, hydrated in gradient alcohol, and antigen-repaired in citrate buffer (pH = 6.0). After that, liver sections were blocked with goat serum at 37 °C and then incubated with primary antibodies, including CD68 (Abcam, Shanghai, China) and F4/80 (Abcam, Shanghai, China), overnight at 4 °C. The sections were incubated with HRP-conjugated antibody, followed by staining with 3,3′-diaminobenzidine (DAB), and counterstained with hematoxylin. After viewing and imaging under a brightfield microscope (DM750, Leica, Shanghai, China), the IHC positive staining areas were quantified using ImageJ software (NIH, Bethesda, MD, USA).

### 2.4. TUNEL Assay

To detect cell apoptosis in liver tissues, liver sections were stained with TUNEL reaction solution using a commercial kit (C1088, Beyotime Biotechnology, Shanghai, China) according to the manufacturer’s guidelines as previously reported [11]. Briefly, paraffin-embedded hepatic tissue sections were dewaxed and hydrated using xylene and gradient alcohol, followed by incubation with 20 μg/mL proteinase K without DNase for 20 min at room temperature, and then incubated with TUNEL reaction solution at 37 °C for 1 h. The sections were washed with PBS 3 times, and counterstained with DAPI (S2110, Solarbio, Beijing, China). The fluorescence signals were observed and photographed using a fluorescence microscope (Axio Observer 5, Carl Zeiss, Jena, Germany).

### 2.5. Dihydroethidium (DHE) Staining

DHE staining was performed to evaluate superoxide production in the liver tissues, as previously described [28]. In brief, the frozen liver tissues, embedded in optimum cutting temperature compound (OCT) were cut into 5 μm sections, then immediately stained with 10 μM DHE (S0063, Beyotime Biotechnology, Shanghai, China) in the dark for 30 min at 37 °C, and then washed with PBS 3 times. Finally, the nuclei were labeled with DAPI (S2110, Solarbio, Beijing, China). The images were acquired using a fluorescence microscope (Axio Observer 5, Carl Zeiss, Jena, Germany).

### 2.6. Plasma Biochemistry and ELISAs

According to previous studies, the levels of alanine transaminase (ALT), aspartate transaminase (AST), and lactate dehydrogenase (LDH) in plasma, and the levels of malondialdehyde (MDA) in the liver were determined using colorimetric assay kits (C009, C010, A020, A003, Nanjing Jiancheng Bioengineering Institute, Nanjing, China) under the manufacturer’s instructions [29]. The hepatic activities of superoxide dismutase (SOD) and glutathione peroxidase (GPX) were examined with commercial kits (A001, A005, Nanjing Jiancheng Bio-engineering Institute, Nanjing, China) according to the manufacturer’s recommendations. The hepatic glutathione (GSH) level was measured using the GSH assay kit (S0053, Beyotime Biotechnology, Shanghai, China), following the manufacturer’s procedure. Plasma TNF-α, IL-1β, MCP-1, IL-6, and RANTES levels were detected using the double-antibody sandwich indirect ELISA kits (EK282, EK201BHS, EK287, EK206, EK2129, MultiSciences Biotech Co., Ltd., Hangzhou, China) according to the manufacturer’s instructions. In brief, plasma samples were separately added into 96-well plates that had been pre-coated with anti-TNF-α, anti-IL-1β, anti-MCP-1, anti-IL-6, and anti-RANTES antibodies, and incubated at room temperature for 90 min. After washing 6 times, the biotinylated anti-TNF-α, anti-IL-1β, anti-MCP-1, anti-IL-6, and anti-RANTES antibodies were respectively applied into plates and incubated for 30 min at room temperature. The HRP-labeled streptavidin was added after washing 6 times, and incubated at room temperature for 30 min. The 96-well plates were further washed 6 times, and incubated with tetramethylbenzidine for 5–30 min at room temperature until the stop solution was added. Finally, optical densities were measured using a SpectraMax Paradigm Multi-Mode microplate Reader (Molecular Devices, Shanghai, China) at 450 nm.

### 2.7. Western Blot Analysis

Western blot analysis was performed as previously reported [30]. Briefly, proteins extracted from liver tissues were separated via SDS polyacrylamide gel electrophoresis and transferred to PVDF membranes (Merck Millipore, Shanghai, China). The transferred membranes were blocked with 5% nonfat milk for 1 h following incubation with primary antibodies against cleaved caspase3, P62, Beclin1, ATG7, LC3B, and GAPDH overnight at 4 °C. The membranes were washed with TBST 3 times, followed by incubation with HRP-labeled secondary antibodies for 1 h at room temperature. Finally, chemiluminescence was visualized using enhanced chemiluminescence reagent (NCM Biotech, Suzhou, China) and imaged using the ChemiDoc XRS system (Bio-Rad, Shanghai, China). All relative protein expression was quantitated using ImageJ software (NIH, Bethesda, MD, USA), and expressed as the values of the target protein band intensity divided by the optical density value of GAPDH. The anti-caspase3 antibody was from Cell Signaling Technology (Shanghai, China). The antibodies against p62, Beclin1, and ATG7 were purchased from Abclonal (Wuhan, China). Anti-LC3B antibody was purchased from Sigma-Aldrich (Shanghai, China). The anti-GAPDH antibody and all secondary antibodies were purchased from Proteintech (Wuhan, China).

### 2.8. Real-Time PCR

Total RNA was extracted from livers or cells using the RNA simple Total RNA Kit (Tiangen, Beijing, China) according to the manufacturer’s instructions. Then, cDNA was synthesized with a FastQuant RT Kit using gDNase (Tiangen, Beijing, China), following the manufacturer’s protocol. Real-time PCR was performed using a SYBR Green kit (Tiangen, Beijing, China) on a CFX connect thermocycler system (Bio-Rad, Shanghai, China) according to the manufacturer’s protocol. The relative mRNA expression levels were calculated based on the comparative threshold cycle (Ct) value, applying the 2^−ΔΔCt^ method, as previously reported [31]. The relative quantification was normalized to GAPDH. The sequences of the primers used are shown in Table 1. 

### 2.9. Cell Culture and Treatment

According to a previous report, the RAW264.7 cells and the AML-12 cells were cultured in DMEM (Hyclone) medium with 10% fetal bovine serum, and maintained in a humidified incubator with 5% CO_2_ at 37 °C [32]. To investigate the effect of Ala-Gln on the LPS-induced inflammatory response, RAW264.7 macrophages were stimulated with LPS (100 ng/mL) for 24 h in the presence or absence of Ala-Gln (10 mM). To determine the role of Ala-Glu on H_2_O_2_-induced oxidative stress, AML-12 cells were treated with H_2_O_2_ (0.3 mM) for 24 h, with or without Ala-Glu (10 mM).

### 2.10. Statistical Analysis

GraphPad Prism (version 8.3.0, La Jolla, CA, USA) was used for data analysis. The data were presented as the mean ± SD. The normality of the data distribution was analyzed using the Shapiro–Wilk test. Statistical differences of the data that followed a normal distribution were assessed using a one-way analysis of variance (ANOVA), followed by the Bonferroni multiple comparison test. Statistical differences of the data that did not follow normal distribution were analyzed using non-parametric tests. The liver injury score data were presented as medians with interquartile range, while the statistical difference was analyzed using Kruskal–Wallis ANOVA, followed by Dunn’s multiple comparisons test. Statistically significance was set as *p* < 0.05.

## 3. Results

### 3.1. Ala-Gln Pretreatment Alleviates Acute Liver Injury in LPS/D-Gal-Treated Mice

**Figure 1:**
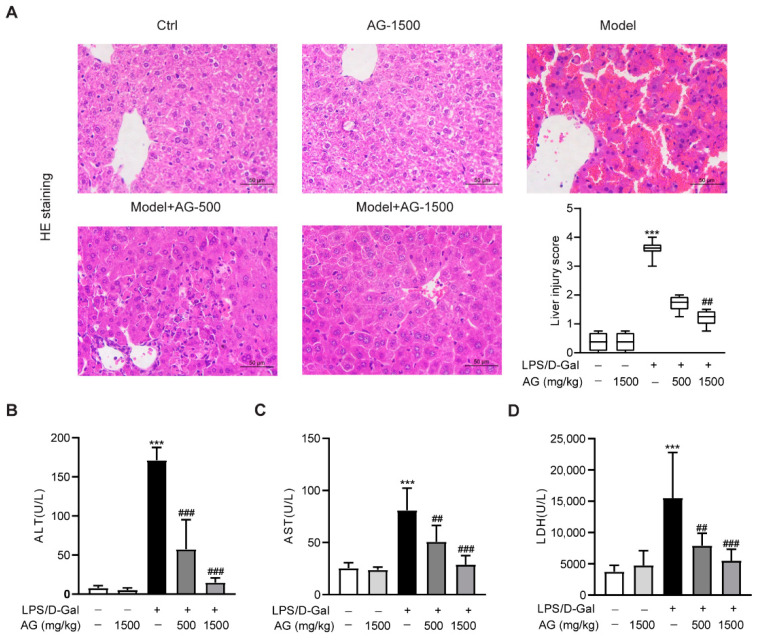
Effects of Ala-Gln on LPS/D-Gal-induced hepatic pathological and biochemical parameters in mice. (**A**) Liver tissues were stained with HE for histopathological analysis (original magnifications, ×400), and the degree of liver injury was scored. (**B**–**D**) plasma ALT, AST, and LDH levels. Data A is presented as medians with interquartile range. Data B–D are expressed as mean ± SD; n = 6–8 in each group. *** *p* < 0.001 vs. vehicle-treated control group, ## *p* < 0.01, ### *p* < 0.001 vs. LPS/D-Gal-treated model group.

To first explore the effect of Ala-Gln on acute liver injury, C57BL6/J mice were intraperitoneally injected with LPS and D-Gal in the presence or absence of different doses of Ala-Gln (500 or 1500 mg/kg). HE staining showed that the mice receiving LPS/D-Gal injection dramatically developed liver damage, while both doses of Ala-Gln pretreatment significantly reduced the degree of liver injury (Figure 1A). Consistently, plasma biochemical assays demonstrated that LPS/D-Gal markedly elevated the levels of ALT, AST, and LDH. However, Ala-Gln greatly attenuated the elevation of ALT, AST, and LDH levels in plasma (Figure 1B–D), which further confirmed the protective effect of Ala-Gln on LPS/D-Gal-induced acute liver injury.

### 3.2. The Protective Effect of Ala-Gln on LPS/D-Gal-Induced Apoptosis in Mice

**Figure 2:**
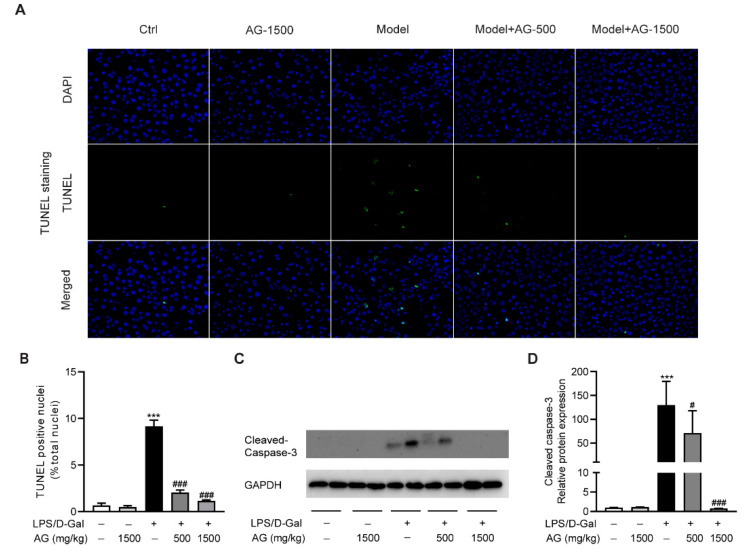
Ala-Gln suppressed apoptosis in LPS/D-Gal-treated mice. (**A**) TUNEL staining (original magnification, ×400) of liver tissues. (**B**) Quantitation of TUNEL-positive cells in liver. (**C**,**D**) Protein expression levels of cleaved-caspase-3 in liver. Data are expressed as mean ± SD, n = 6–8 in each group. *** *p* < 0.001 vs. vehicle-treated control group, # *p* < 0.05, ### *p* < 0.001 vs. LPS/D-Gal-treated model group.

To evaluate the protective effect of Ala-Gln on hepatocyte apoptosis, we examined apoptotic cells in the liver via TUNEL staining. As shown in Figure 2A,B, obvious hepatocyte apoptosis was observed upon LPS/D-Gal stimulation, which was significantly reduced by Ala-Gln pretreatment. Moreover, a Western blot assay showed that the hepatic expression of cleaved caspase-3 was upregulated after LPS/D-Gal injection in mice. However, Ala-Gln markedly attenuated caspase-3 activation, suggesting an antiapoptotic effect by Ala-Gln (Figure 2C,D).

### 3.3. Effect of Ala-Gln on Liver Antioxidant Capacity

**Figure 3:**
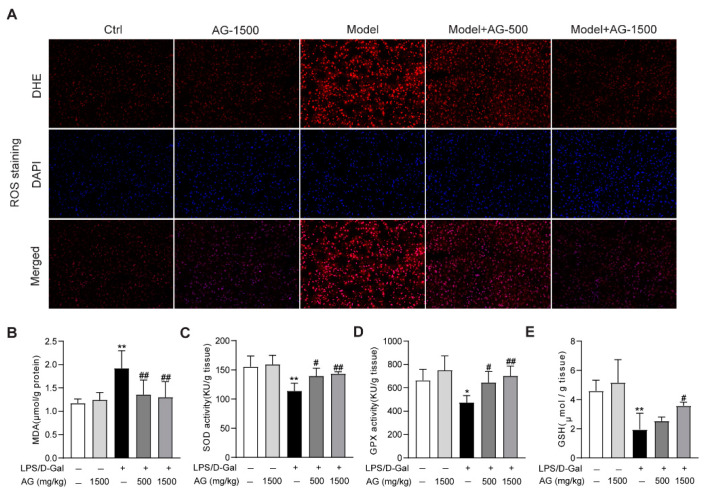
Antioxidant effects of Ala-Gln in liver tissues from mice that received LPS/D-Gal injection. (**A**) ROS detection via DHE staining (original magnification, ×200) in liver tissues. **(B**) Hepatic MDA levels. (**C**) Hepatic SOD activities. (**D**) Hepatic GPX activities. (**E**) Hepatic GSH levels. Data are expressed as mean ± SD, n = 6–8 in each group. * *p* < 0.05, ** *p* < 0.01 vs. vehicle-treated control group, # *p* < 0.05, ## *p* < 0.01 vs. LPS/D-Gal-treated model group.

The apoptosis observed in liver tissue during acute liver injury may result from ROS accumulation in the liver [33]. Thus, we evaluated whether ROS was enriched in the liver tissues from LPS/D-Gal-treated mice. DHE staining indicated that ROS was highly produced, while Ala-Gln pretreatment significantly suppressed ROS production (Figure 3A). Meanwhile, the hepatic oxidative stress caused by LPS/D-Gal stimulation also presented as increased MDA levels, reduced the hepatic activity levels of SOD, GPX, and GSH. However, Ala-Gln administration greatly decreased MDA levels, and elevated SOD and GPX activities, and increased GSH levels in the liver (Figure 3B–E).

### 3.4. Ala-Gln Regulates the Distribution of Hepatic F4/80- and CD68-Positive Immune Cells in the Liver and Attenuates Inflammation

**Figure 4:**
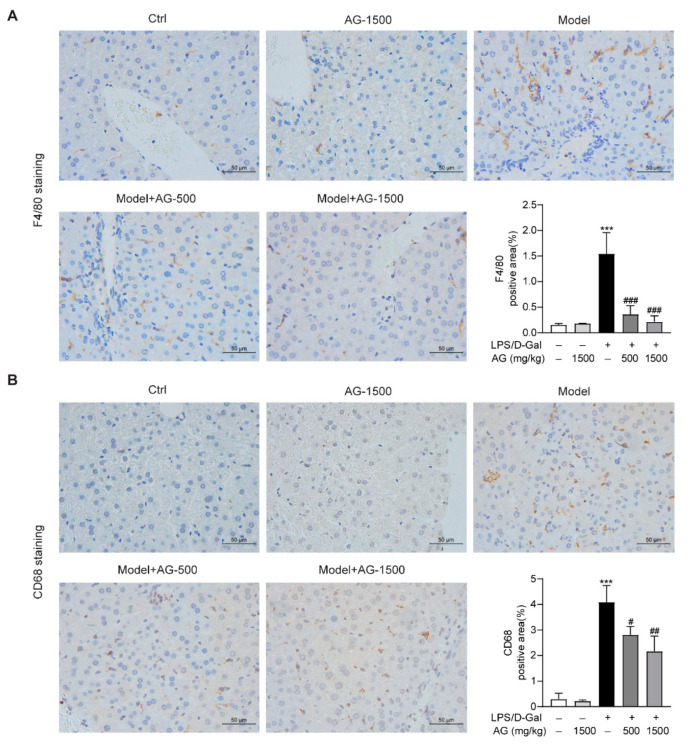
IHC analysis results showing the levels of F4/80 and CD68 in liver tissues in mice. (**A**) IHC staining of F4/80 of liver tissues (original magnifications, ×400), and quantitation of F4/80 positive area. (**B**) IHC staining of CD68 of liver tissues (original magnifications, ×400), and quantitation of CD68 positive area. Data are expressed as mean ± SD, n = 6–8 in each group. *** *p* < 0.001 vs. vehicle-treated control group, # *p* < 0.05, ## *p* < 0.01, ### *p* < 0.001 vs. LPS/D-Gal-treated model group.

Previous studies have reported that hepatic macrophages, which mainly express F4/80 or CD68, are responsible for the aggression of acute liver injury [34]. To investigate whether Ala-Gln has a potential role in the regulation of hepatic macrophages in LPS/D-Gal-induced hepatotoxicity, we detected the expression of F4/80 and CD68 in liver tissues via IHC assays. The results demonstrated that Ala-Gln decreased F4/80 and CD68 levels in a dose-dependent manner, in comparison with LPS/D-Gal treated mice (Figure 4). 

**Figure 5:**
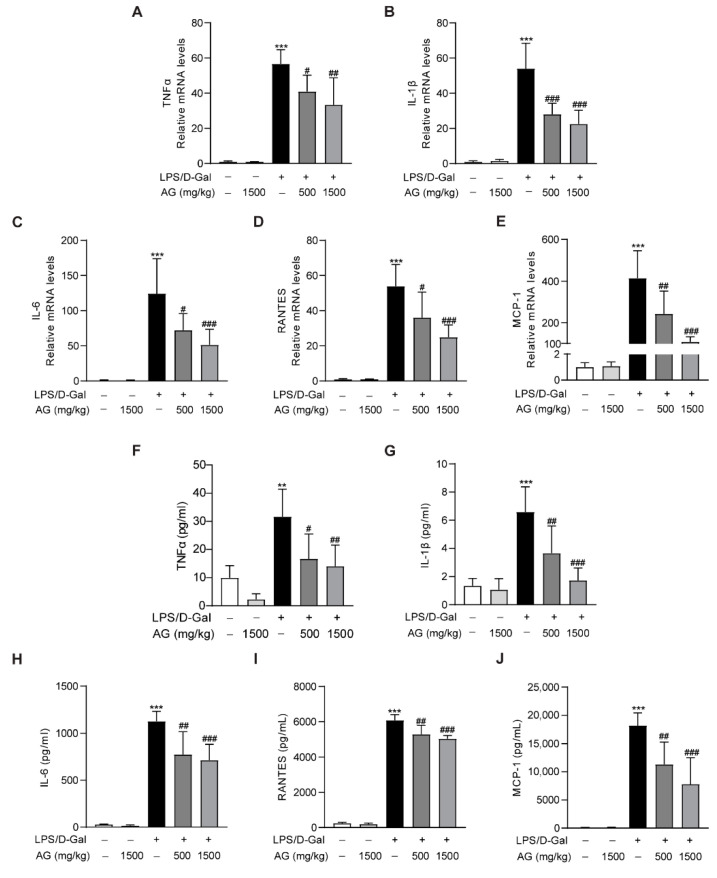
Impact of Ala-Gln on LPS/D-Gal-induced inflammatory responses in liver and plasma. (**A**–**E**) mRNA expression levels of TNF-α, IL-1β, IL-6, RANTES, and MCP-1 in liver tissues. (**F**–**J**) Protein levels of TNF-α, IL-1β, IL-6, RANTES, and MCP-1 in plasma. Data are expressed as mean ± SD, n = 6–8 in each group. ** *p* < 0.01, *** *p* < 0.001 vs. vehicle-treated control group, # *p* < 0.05, ## *p* < 0.01, ### *p* < 0.001 vs. LPS/D-Gal-treated model group.

In addition, real-time PCR showed that the proinflammatory cytokines and chemokines, including TNF-α, IL-1β, IL-6, MCP-1, and RANTES, were significantly increased after LPS/D-Gal treatment for 5 h, while Ala-Gln pretreatment markedly reduced the expression of these proinflammatory factors (Figure 5A–E). Similarly, ELISA assays also indicated a significant upregulation of plasma proinflammatory cytokines in mice that had been injected with LPS/D-Gal, while Ala-Gln dramatically decreased these elevations (Figure 5F–J).

### 3.5. Ala-Gln Promotes Autophagy in the Livers of LPS/D-Gal-Treated Mice

**Figure 6:**
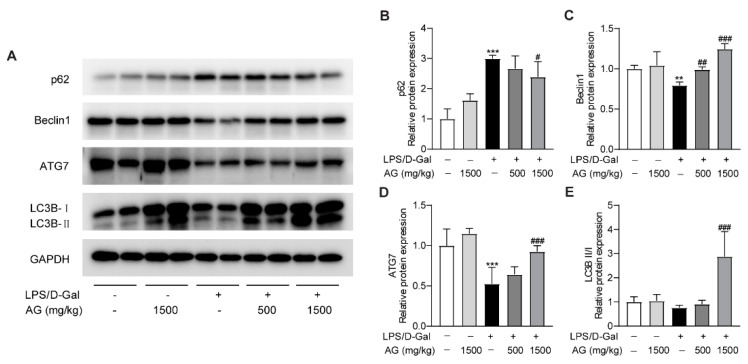
Effect of Ala-Gln on autophagy in liver in LPS/D-Gal-treated mice. (**A**) Protein expression levels of p62, Beclin-1, ATG-7, and LC3B in liver were assessed using Western blot. (**B**–**E**) Quantitation of p62, Beclin-1, ATG-7, and LC3B-II/I expression. Data are expressed as mean ± SD, n = 6–8 in each group. ** *p* < 0.01, *** *p* < 0.001 vs. vehicle-treated control group, # *p* < 0.05, ## *p* < 0.01, ### *p* < 0.001 vs. LPS/D-Gal-treated model group.

A growing number of studies have revealed that autophagy could modulate the pathophysiological process of liver injury [35,36]. To determine whether Ala-Gln affects autophagy during the progress of acute liver injury, we examined the protein expression levels of p62, Beclin-1, ATG-7, and LC3B. The data showed that p62 expression was greatly elevated, while the protein levels of Beclin-1 and ATG-7 were markedly reduced after LPS/D-Gal treatment, which indicated that LPS/D-Gal dysregulated the normal process of autophagy in the liver. However, Ala-Gln pretreatment in LPS/D-Gal-treated mice prevented the increased expression of p62, and enhanced the expression of Beclin-1, ATG-7, and LC3B-II/LC3B-I (Figure 6). The above findings demonstrated that Ala-Gln could modulate autophagy during the LPS/D-Gal-induced progression of acute liver injury.

### 3.6. Ala-Gln Inhibits LPS-Induced Inflammatory Response in RAW264.7 Cells

**Figure 7:**
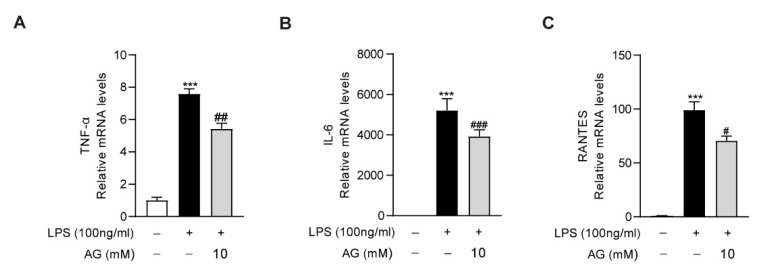
Impact of Ala-Gln on LPS-induced inflammatory responses in RAW264.7 cells. (**A**–**C**) mRNA expression levels of TNF-α, IL-6, and RANTES in RAW264.7 cells. Data are expressed as mean ± SD. *** *p* < 0.001 vs. vehicle-treated control group, # *p* < 0.05, ## *p* < 0.01, ### *p* < 0.001 vs. LPS-treated model group.

As the key role of macrophages in the aggravation of liver inflammation during acute liver injury, we further evaluated the anti-inflammatory effect of Ala-Gln in RAW264.7 cells. We found that LPS treatment significantly increased the expression of proinflammatory cytokines, including IL-6, TNF-α, and RNATES. However, the high degree of expression of these inflammatory factors was markedly downregulated via Ala-Gln treatment, which confirmed that Ala-Gln could directly inhibit the inflammatory response targeting macrophages (Figure 7).

### 3.7. Ala-Gln Suppresses H_2_O_2_-Induced Oxidative Stress in AML-12 Cells

**Figure 8:**
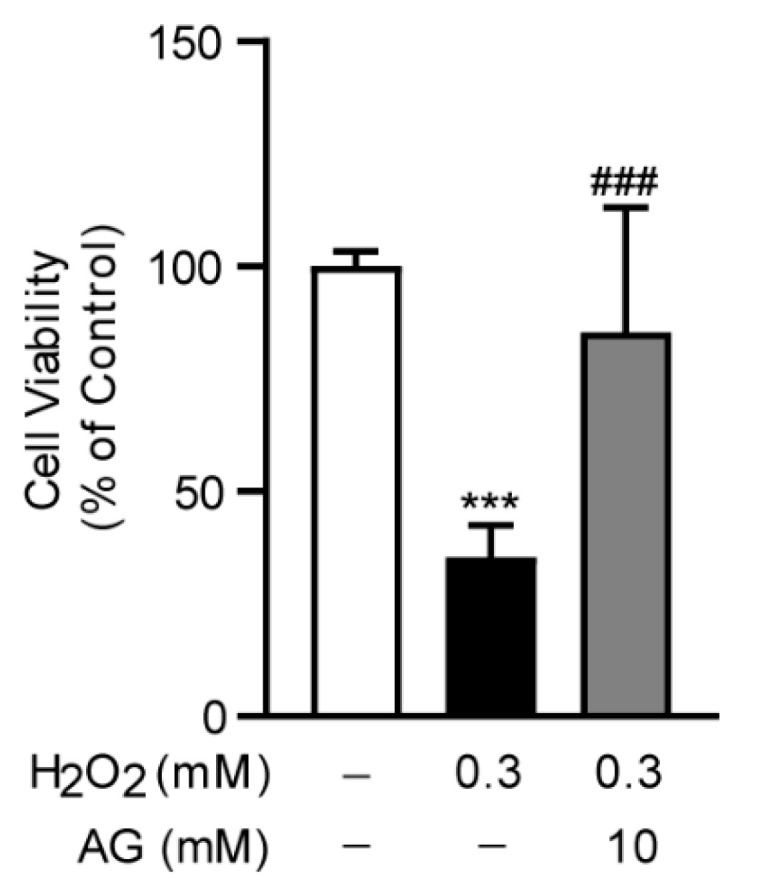
Effect of Ala-Gln on H_2_O_2_-induced oxidative stress in AML-12 cells. Cell viability of AML-12 cells that had received H_2_O_2_ stimulation (0.3 mM) and Ala-Glu treatment (10 mM). Data are expressed as mean ± SD. *** *p* < 0.001 vs. vehicle-treated control group, ### *p* < 0.001 vs. H_2_O_2_-treated model group.

To investigate the anti-oxidative stress effect of Ala-Gln in vitro, we established an oxidative stress model in AML-12 cells through the stimulation of H_2_O_2_, which was frequently used to trigger oxidative stress in cells. Interestingly, we found that Ala-Glu significantly reduced the H_2_O_2_-induced cell toxicity, indicating that the antioxidant effect of Ala-Glu in vivo might be attributable to the chemical properties of Ala-Glu (Figure 8).

## 4. Discussion

Acute liver injury is a challenging clinical problem caused by various etiologies such as drug overdose, virus infection, alcohol abuse, and sepsis, and it is associated with high morbidity and mortality due to the lack of efficient therapies [2]. LPS/D-Gal-induced fulminant hepatitis, which is mainly mediated by an inflammatory response and characterized by massive hepatic apoptosis, is a widely accepted rodent model for investigating the mechanisms and screening protective agents that are related to acute liver injury [11]. In the present study, we found that Ala-Gln significantly improved histological changes in liver and alleviated the levels of liver dysfunction indicators in plasma, including ALT, AST, and LDH. In addition, Ala-Gln suppressed hepatic apoptosis via the reduction of the expression of cleaved capase-3, and inhibited hepatic ROS accumulation through the upregulation of SOD and GPX activities, as well as of GSH levels. Importantly, Ala-Gln markedly reduced the number of intrahepatic macrophages, and it accordingly suppressed the production of proinflammatory cytokines. These data indicated a protective effect of Ala-Gln in LPS/D-Gal-induced acute liver damage, which was associated with the inhibition of oxidative stress and inflammation. Furthermore, the limited autophagic flux following LPS/D-Gal stimulation was restored in the liver after Ala-Gln treatment, as indicated by reduced p62 levels and the increased expression of Beclin-1, ATG-7, and LC3B-II/I, which are consistent with previous reports that the regulation of autophagy is involved in the progression of acute liver damage.

Accumulating evidence has shown that massive hepatocyte apoptosis appeared during the pathogenetic process of acute liver injury [37,38]. Similarly, we also found a large number of apoptotic hepatocytes after LPS/D-Gal stimulation in mice, while Ala-Gln pretreatment significantly alleviated the degree of apoptosis in the liver, as shown by a reduction in the percentage of TUNEL-positive cells and the levels of cleaved caspase-3. Our current findings are consistent with those of other studies regarding the antiapoptotic effects of Ala-Gln against tissue injuries [16,39].

Oxidative stress is the disorder of the redox balance between the accumulation of ROS and the antioxidative system in cells and tissues, which could result in cell damage and tissue injury [40]. Overproduced ROS is a leading cause for the development of oxidative stress, and it aggravates the progress of acute liver failure [3]. The antioxidant effects of Ala-Gln have been proven in skeletal muscle, intestine, and liver in various disease models, in vivo or in vitro [17,41,42]. It has also been reported that Ala-Gln pretreatment could alleviate ischemia/reperfusion-induced oxidative stress in liver transplantation patients, and reduce metabolomic markers of oxidative stress in peritoneal dialysis patients [43,44]. In this study, it was found that Ala-Gln markedly decreased ROS and MDA levels, significantly increasing SOD and GPX activities. SOD is one of the most important antioxidant enzymes involved in ROS elimination. GPX is also an important member of the antioxidant defense system for inhibiting oxidative stress via catalyzing the reduction of hydroperoxides [45]. In addition, we examined the effect of Ala-Glu on the expression levels of SOD1/2 and GPX1–4 in the livers, while no significant effect of Ala-Glu was found on the hepatic expression of these antioxidant genes, suggesting that the role of Ala-Glu on the elevation of SOD and GPX activities might not be dependent on the transcriptional regulation of SOD and GPX (Figure S1). Our data are also consistent with earlier studies demonstrating that Ala-Gln administration significantly increases hepatic levels of GSH [46]. Moreover, the in vitro data for AML-12 cells in the present study indicated that the antioxidant effect of Ala-Glu in vivo might be attributable to the chemical properties of Ala-Glu. Thus, our findings unveil the antioxidant effects of Ala-Gln in the livers of LPS/D-Gal-treated mice, and in H_2_O_2_-treated hepatocytes.

Macrophages are important leukocytes that are involved in host immune defense. Importantly, the abundant proinflammatory factors that are produced by macrophages may also contribute to various acute or chronic diseases [47]. LPS/D-Gal-induced acute liver injury is closely associated with the inflammatory response in intrahepatic macrophages [12]. LPS could induce the abundant expression and release of proinflammatory mediators that contribute to hepatocyte damage and cause a more severe inflammatory response [48]. The present of D-Gal makes intrahepatic macrophages and hepatocytes more sensitive to LPS and LPS-induced inflammatory factors [11,12]. Therefore, inflammation is an important driving factor in the LPS/D-Gal-induced acute liver injury model, and it is essential to investigate whether Ala-Gln plays an anti-inflammatory role in damaged liver tissue. Gerbils that were pretreated with Ala-Gln showed a reduced expression proinflammatory factors, including TNF-α, IL-6, and NF-κB, in a model of brain ischemia–reperfusion injury [49]. A previous study showed that Ala-Gln administration significantly inhibited the production of TNF-α and TNF-α-mediated NF-κB pathway activation in skeletal muscle in hindlimb immobilization-induced disuse muscle atrophy rats [41]. In addition, Cruzat et al. have reported that Ala-Gln could exert protective effects in pancreatic β-cells, that had suffered stimulation from inflammatory mediators derived from macrophages [50]. Our present study confirms that the hepatoprotective role of Ala-Gln in LPS/D-Gal-treated mice may be related to its significant anti-inflammatory effects via the inhibition of the activation of macrophages and the suppression of the production of proinflammatory agents. Whether Ala-Gln has a direct anti-inflammatory effect on macrophages has not been fully investigated. A previous study has shown that Ala-Gln could enhance cell viability and inhibit the expression of inflammatory mediators in LPS-stimulated bovine intestinal epithelial cells [51]. Here, in LPS-treated RAW264.7 cells, we found that Ala-Gln significantly reduced the elevated expression of IL-6, TNF-α, and RANTES, indicating the direct anti-inflammatory effects of Ala-Gln in macrophages.

Autophagy is an evolutionarily conserved physiological process in eukaryotic cells that mediates the degradation of misfolded proteins, damaged organelles, and superfluous molecules as a natural recycling system [35]. The presence of basic autophagy is an important physiological activity for maintaining a steady cellular state. More importantly, the activation of autophagy has a protective effect against multiple tissue injuries under various stress conditions [52]. Numerous studies have indicated that the activation of autophagy could alleviate the progression of liver injury resulting from a variety of stimulations, including LPS, D-Gal, acetaminophen (APAP), concanavalin A (Con A), cholestasis, and hepatic ischemia-reperfusion injury [7,52,53]. Consistent with previous reports, our present study identified decreased autophagy in mice received LPS/D-Gal stimulation [35,36]. Meanwhile, we also uncovered that Ala-Gln could promote p62 degradation, LC3B-II/I, Beclin1, and ATG7 upregulation in the liver from LPS/D-Gal-treated mice. These findings revealed that Ala-Gln could regulate autophagy in the injured liver. However, the evidence surrounding the effect of Ala-Glu on the modulation of autophagy in our present study is still limited. To determine whether Ala-Glu could promote or suppress autophagy, in vitro studies involving an autophagic flux assay using primary hepatocytes or a hepatocyte cell line are needed in the future [54].

## 5. Conclusions

In summary, our results indicated the protective effects of Ala-Gln in LPS/D-Gal-induced liver injury. Furthermore, we elucidated that Ala-Gln could inhibit oxidative stress by upregulating hepatic GSH levels during the development of acute liver injury. In addition, Ala-Gln-induced suppression of inflammation and regulation of autophagy, are also contributors to the improvement of acute liver injury induced via LPS/D-Gal treatment.

## Figures and Tables

**Table 1:** Sequences of primers.

Genes	Gene Accession Number	Forward Primer	Reverse Primer
TNF-α	NM_013693	CAGGCGGTGCCTATGTCTC	CGATCACCCCGAAGTTCAGTAG
IL-6	NM_031168	AATTAAGCCTCCGACTTGTGAAG	CTTCCATCCAGTTGCCTTCTTG
IL-1β	NM_008361	GAAATGCCACCTTTTGACAGTG	TGGATGCTCTCATCAGGACAG
MCP-1	NM_011333	TTAAAAACCTGGATCGGAACCAA	GCATTAGCTTCAGATTTACGGGT
RANTES	NM_013653	GCTGCTTTGCCTACCTCTCC	TCGAGTGACAAACACGACTGC
GAPDH	NM_008084	CGGTTCCGATGCCCTGAGGCTCTT	CGTCACACTTCATGATGGAATTGA

## Data Availability

Data is contained within the article.

## Supplementary Materials

The following supporting information can be downloaded at: https://www.mdpi.com/article/10.3390/antiox11061070/s1, Figure S1: The expression of antioxidant genes in liver samples after LPS/D-Gal stimulation and Ala-Glu treatment.
