# Supplementary material for: Alanyl-Glutamine Protects against Lipopolysaccharide-Induced Liver Injury in Mice via Alleviating Oxidative Stress, Inhibiting Inflammation, and Regulating Autophagy
Source: Antioxidants (Basel). 2022 May 27;11(6):1070. doi: 10.3390/antiox11061070 (PMC9220087; doi:10.3390/antiox11061070)
Supplement: Supplementary file 1 [file antioxidants-11-01070-s001.zip › antioxidants-1716965-supplementary.pdf]

Supplementary Material

Supplementary Figure S1

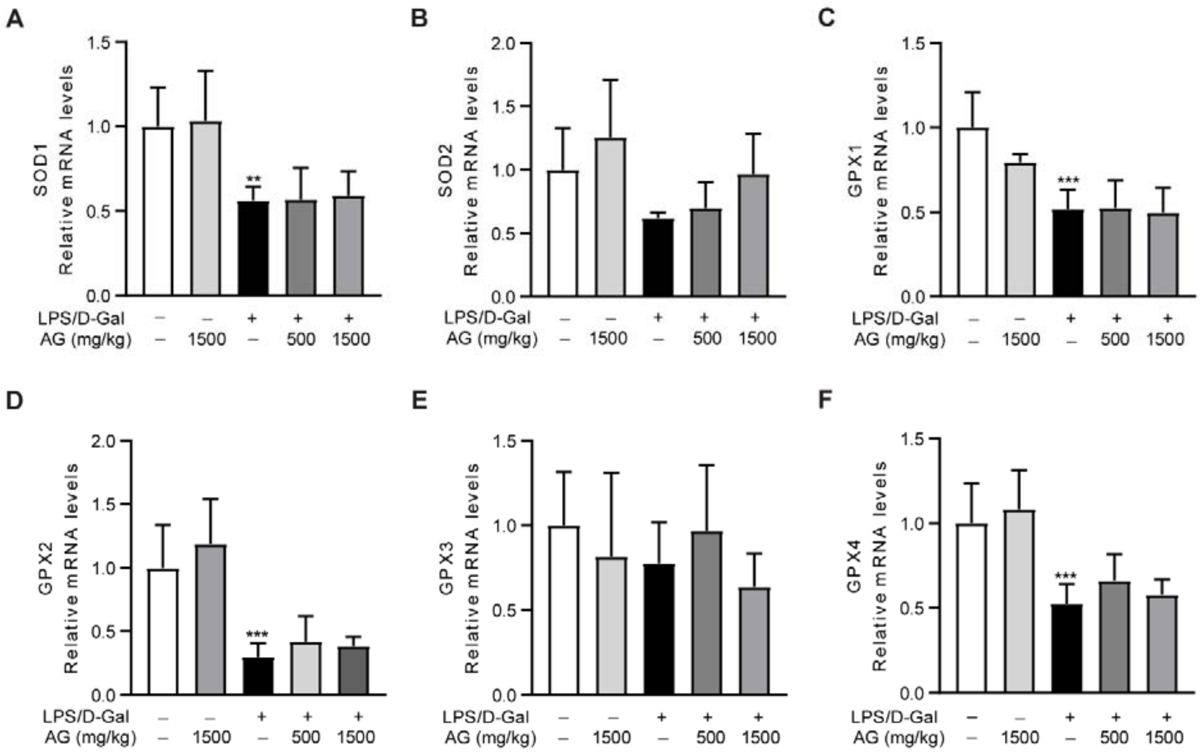

Figure S1. The expression of antioxidant genes in liver samples after LPS/D-Gal stimulation and Ala-Glu treatment. Hepatic mRNA levels of (A) SOD1, (B) SOD2, (C) GPX1, (D) GPX2, (E) GPX3, and (F) GPX4 analyzed by Real-time PCR. Data represent the mean ± SD, n = 6-8 in each group. ** $P < 0.01$, *** $P < 0.001$ vs vehicle-treated control group.
